# Investigating the impact of video inpatient medication education on patient experience and readmission rate: A retrospective observational cohort study with group comparison

**DOI:** 10.1016/j.rcsop.2026.100861

**Published:** 2026-10-07

**Authors:** Courtenay R. Bruce, Ghalib A. Abbasi, Ashleigh Kamencik-Wright, Terrell L. Williams, John Osborn, Janet Reha, Claudia Garcia, April Romero, Richard Eke, Tariq Nisar, Farzan Sasangohar, David Putney

**Affiliations:** a System Patient Experience; Houston Methodist System; 6550 Fannin St., Houston, TX 77030, USA; b Houston Methodist Pharmacy; Houston Methodist System; 6550 Fannin St., Houston, TX 77030, USA; c System Quality Analytics; Houston Methodist System; 6550 Fannin St., Houston, TX 77030, USA; d Guest Relations and Patient Experience; Houston Methodist Baytown Hospital; 4401 Garth Rd, Baytown, TX 77521, USA; e Guest Relations and Patient Experience; Houston Methodist Clear Lake; 18300 Houston Methodist Dr, Houston, TX 77058, USA; f Center for Health Data Science & Analytics, Houston Methodist Hospital, 6550 Fannin St., Houston, TX 77030, USA; g Center for Critical Care, Houston Methodist Hospital, 6550 Fannin St., Houston, TX 77030, USA; h Industrial and Systems Engineering, Texas A&M University, 3131 TAMU, College Station, TX 77843, USA; i Houston Methodist Hospital Pharmacy; Houston Methodist Hospital; 6550 Fannin St., Houston, TX 77030, USA

**Keywords:** Medication, Education, Pharmacy, Text messaging

## Abstract

**Background:**

Effective medication communication during hospitalization is essential for improving patient understanding, medication adherence, and long-term clinical outcomes. Although pharmacist-led medication education has demonstrated benefits, traditional face-to-face counseling is resource-intensive and difficult to scale. We evaluated a scalable, video-based medication education program in which short medication videos were shared with inpatients via SMS text message across a large healthcare system.

**Methods:**

We conducted a retrospective observational study of adult inpatients across seven hospitals within a large quaternary healthcare system. From January 1 through December 31, 2024, 18,391 patients received 232,957 medication education videos via SMS during hospitalization. Outcomes included Hospital Consumer Assessment of Healthcare Providers and Systems (HCAHPS) scores and 30-day hospital readmission rates. Outcomes were compared between patients who viewed at least 50% of assigned videos and those who viewed none. Viewing was self-selected rather than randomly allocated. Engagement patterns were also examined by age, sex, race, ethnicity, and admission type.

**Results:**

Patients who viewed at least 50% of assigned medication education videos had significantly higher HCAHPS scores and significantly lower 30-day readmission rates than patients who did not view any videos. Engagement with the intervention varied across demographic and clinical subgroups, including age, sex, race, ethnicity, and admission type.

**Conclusions:**

A scalable video education program delivered via SMS was associated with improved patient experience and lower hospital readmission rates across a large, multisite healthcare system. These findings support the use of digital medication education to complement traditional pharmacist-led counseling and extend its reach. Further research is needed to identify factors influencing patient engagement and to evaluate the intervention using prospective study designs. Because patients self-selected into viewing, these findings describe associations rather than the intervention's effects.

## 1 Introduction

Self-management plays a key role in effective control of chronic diseases and seems to be a major contributor to the future of care.^14^ In such a care regimen, patients and families are often responsible for coordinating and implementing disease management, including medication administration.^20^ Patient adherence to medication instructions affects clinical outcomes and depends on provider, patient, cultural, historical, and healthcare system factors.^14^ Thus, the ability of acute healthcare professionals to effectively teach patients and families, as well as patients' and families' ability to listen, comprehend, and retain medication instructions, is critical for long-term management and positive outcomes.^2^, ^18^

The bulk of research focusing on the effectiveness of medication education has focused on improving adherence and has evaluated outpatient or post-discharge interventions, including recurrent and personalized telephone counseling sessions, altering the medication regimen, having clinical pharmacist consultations for chronic disease co-management, providing incentives to promote adherence, and instituting medication-taking reminders.^11^ Reviews indicate that such interventions have generally had positive outcomes.^19^ However, despite the general recognition of the important role of medication education, our understanding of inpatient interventions has remained stagnant. Interventions to improve inpatient medication education have almost exclusively focused on face-to-face methods, including written handouts and teach-back methods, in which nurses or pharmacists ask patients specific questions to assess gaps in understanding.^3^, ^22^ Meta-analyses have found pharmacist-led interventions to be successful in improving patient understanding and medication adherence, but face-to-face interventions are often costly, require significant time from clinical pharmacists, and are not easily scalable.^13^

Video has been examined as a vehicle for patient education in a number of clinical contexts. A systematic review of video-assisted patient education identified the format in which information is presented and the complexity of the behavior being addressed as factors associated with efficacy and found that patients in audiovisual cohorts demonstrated higher recall.^1^ Trials in single specialties have reported that video education can improve knowledge compared with verbal instruction alone and reduce the clinical resources required to deliver instruction.5., 10. A recent systematic review of patient education videos aimed at medication-related health behaviors, however, identified only 12 eligible studies across four countries, most of which were small, and few were designed to allow the mechanisms of behavior change to be discerned.^21^ Despite the promise shown by the abovementioned research, there is a paucity of research on the impact of medication education during hospitalization on patient satisfaction, quality of care, or care transition indicators. The inpatient setting, in particular, remains largely unexamined.

To address these gaps, we examined a scalable, video-based program in which short medication education videos were shared with inpatients via SMS. This study aimed to (1) examine the association between 232,957 inpatient medication videos texted to inpatients (January–December 2024) and Hospital Consumer Assessment of Healthcare Providers and Systems (HCAHPS) inpatient and observation survey outcomes; and (2) evaluate associations with readmissions for the same patients. By evaluating HCAHPS official and unofficial survey outcomes (i.e., inpatient and observation status surveys) and readmissions across a large system serving thousands of patients, we hope to broaden understanding and the generalizability of inpatient medication interventions. This was an exploratory, retrospective analysis of routinely collected operational data. It was not designed to establish causality, and its findings are therefore reported throughout as associations rather than as effects of the intervention.

## 2 Methods

### 2.1 Study design and outcomes

The study was reviewed by the hospital's review board, which determined that the work would not be considered regulated human subjects research. All study data were aggregated.

A retrospective observational cohort comparison was conducted to analyze the outcomes of all patients who watched one or more of the medication education videos texted to them during and immediately following their inpatient visit between January 2024 and December 2024, and compared their outcomes with patients who did not watch the videos texted to them during the same time and on the same hospital units. Video viewing was captured automatically in the vendor's server logs by an automated script that recorded the proportion of each video played. The 50% threshold refers to the proportion of an individual video's duration that was played. Grouping was carried out at the encounter level rather than the patient level. The underlying video, readmission, and survey data are all held at the encounter level, and the 17,149 records linked to the system quality database were matched on a unique encounter identifier.

Throughout this paper, counts described as patients refer to distinct individuals; all other counts, including survey returns, viewing groups, and the totals beneath each table, refer to encounters, and a patient admitted more than once during the study period contributes more than one to these counts. Each encounter was assigned to one of three mutually exclusive groups: (a) watched 50% or more, where at least one video sent during that encounter was played to at least half of its duration; (b) watched 0–49%, where at least one video was opened but none reached the 50% threshold; and (c) did not watch, where no video sent during the encounter was opened. An encounter in which three videos were sent and one was played for at least half its length was therefore assigned in full to the first group. The two watching groups consist of encounters in which at least one video was sent. The comparison group is wider: it comprises all survey returns in the same period and on the same units other than those in which a video was watched, and therefore includes both encounters in which videos were sent and not opened and encounters in which no video was sent at all. Comparisons in this paper are accordingly between encounters in which a video was watched and all other returns, not between video recipients who watched and video recipients who did not. The vendor has confirmed that the source extract contains one row for every survey response from patients discharged during the study period and is not limited to encounters in which a medication video was sent. Patients were not randomly allocated and chose for themselves whether to open the videos sent to them, so group membership was self-selected. No matching or weighting was applied to reduce the resulting imbalance between groups; comparisons rest on multivariable adjustment alone.

Our primary outcome measures were patient experience scores and readmissions. Patient experience was defined as patients' subjective evaluation of all interactions they have with the healthcare system across the continuum of care, often shaped by organizational culture.^16^ Patient experience was measured using the HCAHPS survey (Appendix A). We treated HCAHPS scores as continuous rather than ordinal because of the large sample size. Ordinal scales become stable with large data. An ordinal scale also forces responses into fixed buckets that may not reflect the full range of differences in patient experience. While we focused on *Communication about Medicines* and summative dimensions such as *Overall Hospital Rating* and *Recommendation of Hospital*, we report results for all dimensions here.

Consistent with the inclusion and exclusion criteria set by the Centers for Medicare & Medicaid Services^4^ methodological framework, readmission rates were defined as any subsequent unplanned inpatient admission to any of our acute care facilities within 30 days of hospital discharge. Unplanned, any-cause, acute-care inpatient admissions were evaluated, excluding transfer encounters. Where an encounter was followed by more than one admission, the first admission in the 30-day window following discharge was counted. A patient admitted on several occasions during the study period could therefore contribute more than one encounter, and more than one survey response, to the analysis.

### 2.2 Setting and context

The study was conducted in a quaternary medical system in Texas. The system consists of one academic medical center (>1386 beds) and, at the time of this study, six community hospitals, ranging from approximately 178 to 500 beds. The sample size was determined by the number of patients admitted during the study implementation period, and only patients aged 18 years or older were included.

### 2.3 Education and training for pharmacists and nurses

Nurses used a semi-scripted guide from a trained patient experience team member to answer patients' questions about the medication videos. The task force conducted two virtual sessions with acute care nurse leaders to raise awareness of the types of questions patients may ask about the videos and to explain the workflow for routing alerts to the pharmacy. The training was standardized using PowerPoint teaching material.

Pharmacists were taught by two pharmacy co-authors of this paper (GA, DP) how to receive notifications, where and how to identify the patient who wanted to speak with a pharmacist, and how to adapt the conversation based on the patient's query.

### 2.4 Medication video protocol

As part of the standard of care, medication education was conducted by bedside nursing and reinforced by pharmacists, as indicated by nursing staff. Patient education was documented in the electronic health record (EHR) nursing flowsheets and pharmacists' progress notes, which allow customization of medication names, purposes, side effects, and health monitoring.

For the intervention cohort,^12^ a nursing and patient education vendor, supplied 150 2–3-min medication education videos for this study, as well as 6 30-s medication videos generated by pharmacy staff. At every hospital admission, registration staff asked patients whether they consented to receive healthcare updates via text message. Once consent was provided, the administrative staff entered the patients' and family members' phone numbers in the EHR. CareSense was the vendor used to deliver text messages containing medication videos during and after the hospital stay.

A participatory design method was used to design and review the text messages and the medication education videos. The initial set of text messages was developed iteratively by a task force comprising three pharmacists, three nurses, and four patient experience representatives from different hospitals within the system. We also drew on feedback from patient partners who were recently hospitalized.

Patients were included in the intervention cohort if they were started on at least one new medication during their hospital stay, i.e., medications not included on the admission medication list for any previous hospitalizations. The first automated message was triggered when the patient reached inpatient bed status and a nurse scanned a new medication into the medication administration record (MAR) in the EHR. This would then trigger the text message and video link to be sent to the patient. The content of these videos focuses on education about the purposes and side effects of each new medication administered. The videos were specific to the medication that was scanned in the EHR, and the text message specified the number of medications (Fig. 1). (See Fig. 2.)

**Fig. 1:**
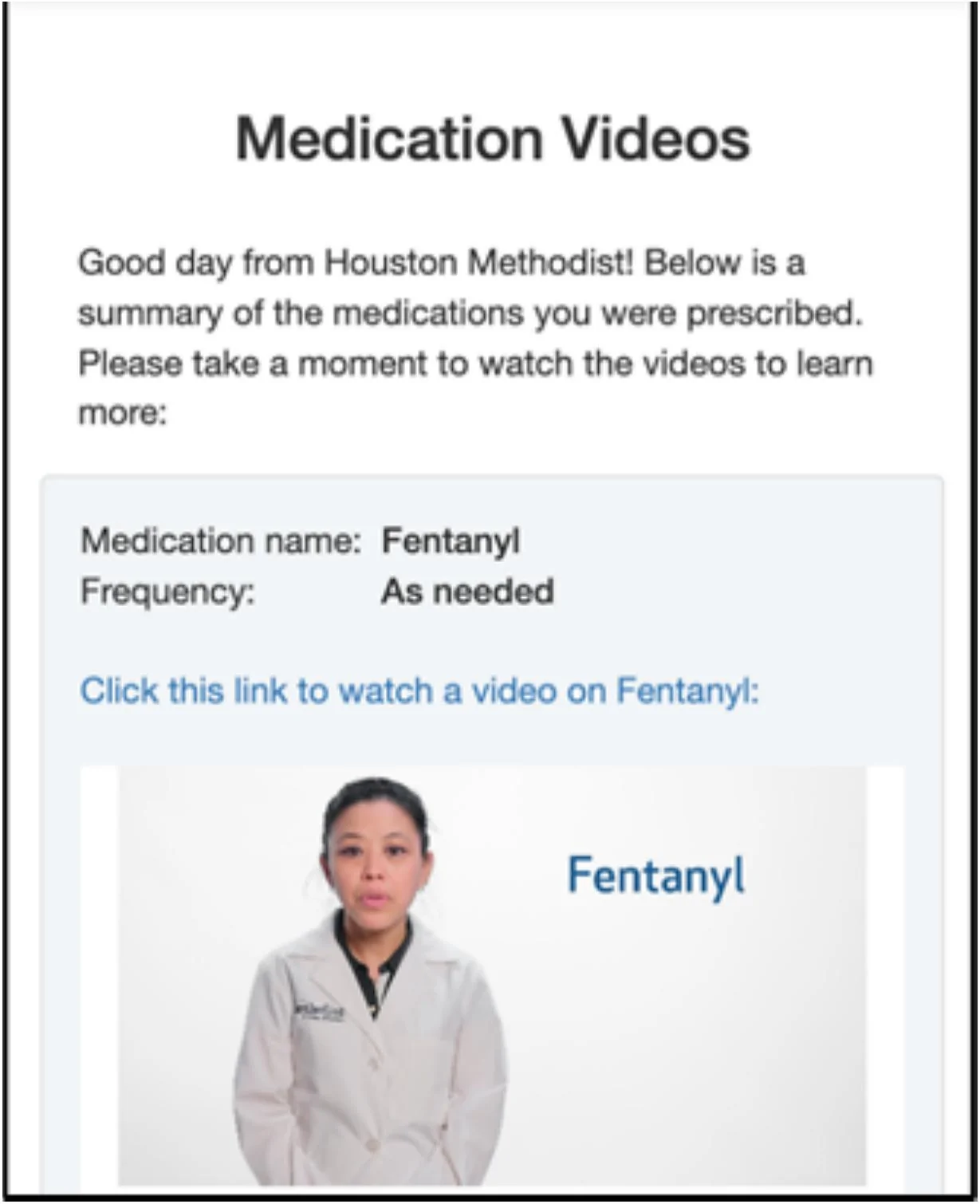
A sample screen from a medication video. Hello. I'm Dr. Kathryn Pidcock and I'm one of the pharmacists here at [removed for blind review] Hospital. Today, I would like to talk to you about your medication, Fentanyl. This medication is used to ease pain. The most common side effects that you might experience are constipation, slow breathing, dizziness, and feeling confused. You should avoid driving and doing other tasks or actions that call for you to be alert until you see how this drug affects you. Please ask your nurse, doctor, or pharmacist any questions that you might have. [removed for blind review] thanks you for your time.

**Fig. 2:**
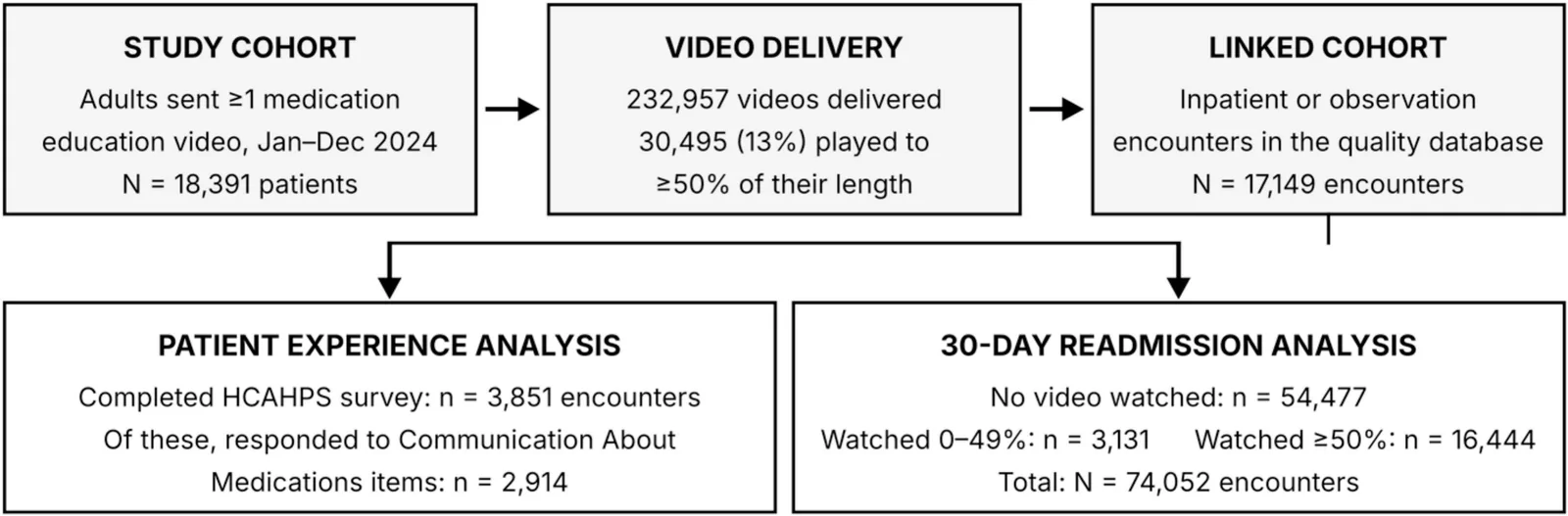
Participant flow through the study and analytic cohorts. HCAHPS, Hospital Consumer Assessment of Healthcare Providers and Systems.

If the nurse scanned a medication without a video on file, no text message was sent. The videos were available in English, Spanish, Arabic, and Vietnamese, which patients could toggle to their preferred language.

We held the dissemination of the videos for 1 h after the nurse scanned medications to aggregate all videos together for any new medications so that the patients would only receive one text message, which was limited to a total of two text messages during the entire hospitalization, to limit the potential for overwhelming the patient with text messages.

A transcription, including bullet points of key components, accompanied the video for patients who preferred written material. At the end of the video, patients were asked whether they had any questions they would like to discuss with a pharmacist. If they clicked “yes” to indicate they had questions for a pharmacist, we routed an alert by phone or email to the pharmacist assigned to that unit so they could speak with the patients in person. A closed-loop feedback structure was implemented to ensure the pharmacist addressed patient concerns within a few hours of the notification during each facility's established business hours of 9:00 a.m. to 6:00 p.m. Requests submitted outside those hours were answered the following morning, and patients were told to expect this on the same screen they used to request the call back.

### 2.5 Analysis methods

The primary independent variable was whether the video was watched, coded as binary (watched = 1, not watched = 0). Readmissions were classified as binary (1 = yes vs 0 = no). A multivariate generalized logistic regression model was built for this binary outcome. The multivariate model included patients' demographics (age, gender, race, ethnicity), admission type, and the Charlson Comorbidity Index (CCI). Data were extracted from CareSense and then imported into a secure Structured Query Language (SQL) server. From the SQL server, the CareSense data were joined to the EHR database, where readmission data are stored. Once the databases were scrubbed for duplicates, the data were imported into Tableau to facilitate calculations of distinct encounter counts.

HCAHPS domain scores were calculated using top-box scoring: for each item, the number of responses in the most positive category divided by the number of responses to that item, expressed as a percentage. Patients who did not answer a given item were excluded from that item's score, and not every patient answered every item, so the denominator varies by domain. For the HCAHPS, we treated these scores as continuous rather than ordinal, since the overall score patterns stabilize with large datasets. Ordinal scales force responses into fixed categories that may not reflect the full range of differences in patient experience. Hence, reporting mean differences is more sensitive and statistically efficient. Additionally, a multivariable generalized linear regression using the generalized estimating equation (GEE) approach, accounting for repeated measurements, was fitted because our data include repeated patient-level observations across different domains. For the GEE, we used the link function “identity” and the correlation structure to establish independence. The fixed effect is the hospital's dimensions; the repeated effect is at the patient level; and the family is Gaussian. In general, GEE-GLM models are more robust to bias arising from within-subject correlations. GEE also produces more robust standard errors (SE). The multivariate model for HCAHPS was also controlled for patients' demographics (age, gender, race, ethnicity), admission type, and CCI. We have reported odds ratios (OR), estimated differences (Δβ), and 95% confidence intervals (CI). A *p* < 0.05 was considered statistically significant. The model for the readmission outcome was fitted using the same generalized estimating equation approach, with the repeated effect specified for the patient's medical record number, so that encounters from the same patient were treated as clustered rather than independent. The models for all HCAHPS domains (Table 4) and for readmission (Table 6) were fitted on data containing repeated encounters, which is why their group totals exceed the number of distinct survey respondents. The models restricted to the Communication about Medicines dimension (Table 5, Table 7) were fitted on the subset of records for that dimension (total *N* = 8097), in which no encounter is repeated, so that one row represents one encounter. All statistical analyses were performed in R v.4.4.1.

Because viewing was self-selected rather than randomly allocated, the groups differ in observed characteristics. We did not apply propensity score matching or weighting to address this; differences between groups were accounted for by multivariable adjustment for age, gender, race, ethnicity, admission type and CCI alone.

### 2.6 Analysis of free-text patient comments

Free-text comments submitted through the HCAHPS survey (*n* = 715) and through CareSense (*n* = 344) between January and December 2024 were reviewed using a grounded theory approach.^17^ Four reviewers coded the comments independently and resolved discrepancies by consensus. Microsoft Copilot was used to support the initial organization of codes, and all final coding decisions and thematic assignments were reviewed and validated by the study team. Four themes were identified and are reported in the Results. Generative AI was used in this study only for the purpose described above; no other use was made of it in the analysis or in the preparation of this manuscript.

## 3 Results

A total of 18,391 patients received 232,957 medication videos from January 1st to December 31st, 2024, across all 7 hospitals. Of the 232,957 videos sent, 30,495 (13%) were played to at least 50% of their length.

Among patients who watched the videos, 17,149 matched encounters were in the system quality database for inpatient or observation-status encounters during the study period. Of those 17,149 encounters, 3851 yielded a completed HCAHPS survey, and 2914 included a response to the medication questions. A medication video was opened in 40,986 encounters. In 777 of these (1.9%), a patient used the prompt at the end of the video to ask to speak with a pharmacist, and all were addressed and closed by a pharmacist with appropriate follow-up, either within business hours or on the following morning. A patient readmitted later in the study period contributes a separate encounter, so these are not 777 distinct patients. This denominator counts every encounter in which a video was opened and is therefore larger than the viewing groups in the readmission analysis, which additionally require an unplanned admission and a returned survey to be eligible.

### 3.1 Patient demographics

Females were more likely than males to watch the medication video for both inpatient (54% versus 46%) and observation-status patients (58% versus 42%) at every hospital in our system (Table 1). The ages are stratified in Table 2, showing that within the 18–34 age cohort females accounted for 63% of video watchers and males 37%. Most patients (87%) who watched the video were discharged home or home with home health.

**Table 1:** Number (top) and percentage (bottom) of female vs. male video watchers.

Site	Inpatient			Observation			Total video watchers		
	Female	Male	IP total	Female	Male	OBS total	Female	Male	Total
Hospital 1	737	680	1417	360	221	581	1097	901	1998
Hospital 2	425	391	816	189	158	347	614	549	1163
Hospital 3	1611	1456	3067	667	541	1208	2278	1997	4275
Hospital 4	1190	1010	2200	632	407	1039	1822	1417	3239
Hospital 5	346	269	615	97	56	153	443	325	768
Hospital 6	1117	966	2083	590	487	1077	1707	1453	3160
Hospital 7	1100	894	1994	324	228	552	1424	1122	2546
Total	6526	5666	12,192	2859	2098	4957	9385	7764	17,149
Site	Inpatient			Observation			Total video watchers		
	% Female	% Male	% IP total	% Female	% Male	% OBS total	% Female	% Male	% Total
Hospital 1	52%	48%	100%	62%	38%	100%	55%	45%	100%
Hospital 2	52%	48%	100%	54%	46%	100%	53%	47%	100%
Hospital 3	53%	47%	100%	55%	45%	100%	53%	47%	100%
Hospital 4	54%	46%	100%	61%	39%	100%	56%	44%	100%
Hospital 5	56%	44%	100%	63%	37%	100%	58%	42%	100%
Hospital 6	54%	46%	100%	55%	45%	100%	54%	46%	100%
Hospital 7	55%	45%	100%	59%	41%	100%	56%	44%	100%
Total	54%	46%	100%	58%	42%	100%	55%	45%	100%

**Table 2:** Comparison of male vs. female video watchers by age.

Patient age of video watched						
Age	Female	Male	Total	%Female	%Male	Diff female to male
Not assigned/Unknown	4	2	6	67%	33%	33%
18–34	538	319	857	63%	37%	26%
35–49	1079	723	1802	60%	40%	20%
50–64	1986	1781	3767	53%	47%	5%
65–79	3755	3449	7204	52%	48%	4%
80+	2023	1490	3513	58%	42%	15%
Total	9385	7764	17,149	55%	45%	9%

### 3.2 Patient experience

The breakdown of HCAHPS scores is shown in Table 3. The groups in that table were formed in February 2025, before the grouping used in Table 4, Table 5, Table 6, Table 7 was settled, and differ from it in two respects. Only two groups were formed: encounters with a returned survey in which a video was watched for at least half its length, and every other returned survey from the same period. Eligibility for that second group was not restricted to the video program, so it contains encounters in which 0–49% of a video was watched as well as encounters in which no video was sent, including some from patients who were never part of the program. Table 4, Table 5, Table 6, Table 7 instead treat encounters watching 0–49% as a group of their own. The two sets of tables therefore do not partition the same population in the same way, and their group totals are not directly comparable. Five of the 7 hospitals (including the large urban hospital) had higher scores on the medication questions in the HCAHPS survey (an average improvement of 3.12 points) when comparing patients who watched the video with those who did not, within the same units/hospitals. Two community hospitals experienced a decline in medication questions (average deterioration of 1.3 points). All hospitals achieved higher scores in the video-watching cohort on at least 5 of the 9 HCAHPS domains. Five hospitals experienced increases in scores across all HCAHPS domains (average: 3.12 points). One hospital improved by an average of 3.5 points in every domain, and another top-performing hospital improved by an average of 4.9 points in every domain, when comparing video watchers to non-watchers.

**Table 3:** HCAHPS top-box domain scores by video viewing group, within hospital. Scores are top-box percentages (0−100) for nine domains at each of the seven study hospitals. Watched: 3851 encounters; did not watch: 25,062. Difference columns are the watched score minus the did-not-watch score, in percentage points, so positive values favor video watchers. The grouping used here differs from that in Table 4, Table 5, Table 6, Table 7; see text.

HCAHPS domain	Hospital 1 watched *n* = 305	Hospital 1 did not watch^a^ (*n* = 1575)	*Diff watched* vs *not watched*	Hospital 2 watched *n* = 359	Hospital 2 did not watch (*n* = 1906)	*Diff watched* vs *not watched*	Hospital 3 watched *n* = 1114	Hospital 3 did not watch (*n* = 8034)	*Diff watched* vs *not watched*
Overall hospital rating	83.9	82.7	*1.2*	85.0	80.4	*4.6*	83.4	79.9	*3.5*
Willing to recommend	87.5	84.1	*3.4*	83.4	78.3	*5.1*	84.8	82.9	*1.9*
Nurse communication	87.3	85.6	*1.7*	85.7	84.0	*1.7*	81.2	79.6	*1.6*
Doctor communication	82.4	80.7	*1.7*	82.2	79.6	*2.6*	85.5	83.0	*2.5*
Care transition	65.9	62.8	*3.1*	63.0	58.3	*4.7*	64.2	61.5	*2.7*
**Communication about medication**	**69.1**	**66.4**	***2.7***	**73.0**	**67.3**	***5.7***	**62.2**	**61.8**	***0.4***
Discharge information	90.7	90.3	*0.4*	90.0	89.7	*0.3*	89.7	88.7	*1.0*
Responsiveness	76.9	74.1	*2.8*	74.1	70.6	*3.5*	65.7	62.2	*3.5*
Cleanliness	78.0	79.7	*−1.7*	87.3	83.6	*3.7*	77.4	74.3	*3.1*
HCAHPS domain	Hospital 4 watched *n* = 748	Hospital 4 did not watch (*n* = 3368)	*Diff watched* vs *not watched*	Hospital 5 watched *n* = 197	Hospital 5 did not watch (*n* = 4170)	*Diff watched* vs *not watched*	Hospital 6 watched *n* = 658	Hospital 6 did not watch (*n* = 2736)	*Diff watched* vs *not watched*
Overall hospital rating	83.4	82.5	*0.9*	89.8	84.1	*5.7*	86.9	83.8	*3.1*
Willing to recommend	84.7	83.8	*0.9*	90.7	86.0	*4.7*	87.4	84.9	*2.5*
Nurse communication	81.7	81.3	*0.4*	85.7	82.5	*3.2*	84.8	83.3	*1.5*
Doctor communication	80.3	81.1	*−0.8*	80.7	79.9	*0.8*	83.6	81.9	*1.7*
Care transition	58.7	60.3	*−1.6*	64.3	61.4	*2.9*	65.7	62.6	*3.1*
**Communication about medication**	**61.4**	**63.8**	***−2.4***	**65.1**	**64.5**	***0.6***	**67.6**	**67.8**	***−0.2***
Discharge information	88.0	87.2	*0.8*	91.5	89.2	*2.3*	88.8	89.3	*−0.5*
Responsiveness	63.1	64.1	*−1.0*	63.5	67.5	*−4.0*	69.6	68.7	*0.9*
Cleanliness	83.7	82.5	*1.2*	87.2	84.1	*3.1*	85.8	85.2	*0.6*
HCAHPS domain	Hospital 7 watched *n* = 470	Hospital 7 did not watch (*n* = 3273)	*Diff watched* vs *not watched*						
Overall hospital rating	85.1	77.1	*8.0*						
Willing to recommend	83.8	77.5	*6.3*						
Nurse communication	86.7	81.5	*5.2*						
Doctor communication	82.9	78.9	*4.0*						
Care transition	63.1	59.4	*3.7*						
**Communication about medication**	**69.8**	**63.6**	***6.2***						
Discharge information	91.1	87.5	*3.6*						
Responsiveness	73.2	67.7	*5.5*						
Cleanliness	82.5	79.9	*2.6*						

a Did not watch comprises all survey returns at the time this table was prepared other than those in which a video was watched to at least half its length. It therefore includes returns in which 0–49% of a video was watched and returns from patients who were never sent a video. Membership of this column was not restricted by eligibility for the video program, and the proportion of these returns from patients outside the program cannot be determined from the data as assembled. Where a patient was sent several videos and watched at least one to half its length, that patient's response was counted in the watched column.

**Table 4:** Group comparison results for all HCAHPS domains.

Variables	Δβ	Lower CI	Upper CI	Pr(>|t|)
(Intercept)	76.23	73.59	78.87	<0.001
Group = Not watched	Ref	Ref	Ref	Ref
Group - Watched 0–49%	1.92	−0.1	3.94	0.06
Group = Watched 50% or more	2.19	1.24	3.14	<0.001
Admit age	−0.05	−0.09	−0.02	<0.001
Gender- Male [Female = Ref]	1.4	0.5	2.31	<0.001
Race- White- Ref	Ref	Ref	Ref	Ref
Race- Black	3.64	2.35	4.93	<0.001
Race- Asian	0.15	−2.01	2.31	0.89
Race- Others [Native American + Declined + Unavailable]	−0.11	−2.89	2.67	0.94
Ethnicity - Hispanics [Non-Hispanics = ref]	6.17	4.86	7.48	<0.001
Admit type- [Emergency]	Ref	Ref	Ref	Ref
Admit type- Elective	4.38	3.34	5.42	<0.001
Admit type- Urgent	3.1	0.51	5.69	0.02
Charlson's Comorbidity Index (CCI)	−0.29	−0.4	−0.17	<0.001

^⁎^Total N for the groups in this model: Not Watched = 68,002; Watched – 0 to 49% = 5430; Watched 50% or more = 39,070. These are counts of rows in the model, which contains repeated encounters; the GEE-GLM accounts for the resulting clustering.

**Table 5:** Group comparison for the Communication about Medication domain of HCAHPS.

Variables	Δβ	Lower CI	Upper CI	Pr(>|t|)
(Intercept)	77.95	72.97	82.92	<0.001
Group = Not watched	Ref	Ref	Ref	Ref
Group - Watched 0–49%	3.54	−0.7	7.79	0.1
Group = Watched 50% or more	2.5	0.59	4.4	0.01
Admit age	−0.26	−0.33	−0.2	<0.001
Gender- Male [Female = Ref]	3.83	2.02	5.65	<0.001
Race- White- Ref	Ref	Ref	Ref	Ref
Race- Black	8.68	6.05	11.31	<0.001
Race- Asian	1.4	−2.67	5.47	0.5
Race- Others [Native American + Declined + Unavailable]	0.49	−5.07	6.06	0.86
Ethnicity - Hispanics [Non-Hispanics = ref]	8.2	5.46	10.94	<0.001
Admit type- [Emergency]	Ref	Ref	Ref	Ref
Admit type- Elective	1.24	−1.03	3.51	0.29
Admit type- Urgent	5.73	0.17	11.29	0.04
Charlson's Comorbidity Index (CCI)	−0.27	−0.5	−0.04	0.02

^⁎^Total N for the groups in this model: Not Watched = 4799; Watched – 0 to 49% = 384; Watched 50% or more = 2914; 8097 in total. This model is restricted to records in the Communication about Medicines dimension, where no encounter is repeated, so one row represents one encounter.

**Table 6:** Group comparison analysis for readmission.

Variables	Odds ratio (95% CI)	*P*-values
(Intercept)	0.05 [0.03–0.07]	<0.001
**Group = Not watched**	**Ref**	**Ref**
Group - Watched 0–49%	0.94 [0.7–1.27]	0.70
Group = Watched 50% or more	0.72 [0.62–0.84]	<0.001
Admit age	1 [1–1.01]	0.26
Gender- Male [Female = Ref]	1.03 [0.9–1.17]	0.68
**Race- White- Ref**	**Ref**	**Ref**
Race- Black	1.07 [0.89–1.27]	0.49
Race- Asian	0.93 [0.68–1.26]	0.64
Race- Others [Native American + Declined + Unavailable]	1.39 [0.93–2.07]	0.11
Ethnicity - Hispanics [Non-Hispanics = ref]	1.03 [0.84–1.26]	0.79
**Admit type- [Emergency]**	**Ref**	**Ref**
Admit type- Elective	0.57 [0.47–0.7]	<0.001
Admit type- Urgent	0.57 [0.37–0.89]	0.01
Charlson's Comorbidity Index	1.11 [1.09–1.12]	<0.001

^⁎^Total N for the groups in this model: Not Watched = 54,477 encounters; Watched – 0 to 49% = 3131 encounters; Watched 50% or more = 16,444 encounters; 74,052 encounters in total.

**Table 7:** Multivariable logistic regression analysis of 30-day readmission among patients with available HCAHPS Communication About Medications responses.

Variables	Odds ratio (95% CI)	P-values
(Intercept)	0.04 [0.02–0.07]	<0.001
Group = Not watched	Ref	Ref
Group - Watched 0–49%	1.1 [0.72–1.68]	0.65
Group = Watched 50% or more	0.77 [0.62–0.95]	0.01
Admit age	1 [1–1.01]	0.43
Gender- Male [Female = Ref]	1.12 [0.93–1.36]	0.24
Race- White- Ref	Ref	Ref
Race- Black	0.91 [0.68–1.2]	0.49
Race- Asian	0.88 [0.56–1.36]	0.56
Race- Others [Native American + Declined + Unavailable]	1.47 [0.85–2.57]	0.17
Ethnicity - Hispanics [Non-Hispanics = ref]	0.83 [0.61–1.13]	0.23
Admit type- [Emergency]	Ref	Ref
Admit type- Elective	0.59 [0.44–0.78]	<0.001
Admit type- Urgent	0.73 [0.39–1.36]	0.32
Charlson's Comorbidity Index	1.11 [1.09–1.13]	<0.001

^⁎^Total *N* for the groups in this model: Not Watched = 4799; Watched – 0 to 49% = 384; Watched 50% or more = 2914; 8097 in total. As for Table 5, this model is restricted to records in the Communication about Medicines dimension, where no encounter is repeated, so one row represents one encounter.

The domains that improved the most were overall hospital rating, likelihood of recommending the hospital, responsiveness, and communication about medications. In terms of hospital demographics, the two community hospitals with the most diverse patient populations and the highest case mix indices also experienced the greatest improvements in HCAHPS performance among video-watchers compared to non-watchers. The two hospitals with the fewest HCAHPS improvements have the least diverse patient populations.

As shown in Table 4, the multivariable GEE-GLM regression showed that participants who watched 50% or more had higher HCAHPS scores when all domains were considered compared to those who did not watch at all [Mean = 76.7 vs 74.3; Δβ = 2.19; 95% CI [1.24, 3.14]; *p* < 0.001]. There was no significant difference in the positive score domain between those who watched less than 50% of the videos and those who did not watch at all [Mean = 76.2 vs. 74.3; Δβ = 1.92; 95% CI [−0.10, 3.94]; *p* = 0.06]. In addition, age was a significant predictor of satisfaction. Each additional year of age was associated with a lower coefficient estimate of high satisfaction, with an average difference of −0.05 (95% CI [−0.09, −0.02]; *p* < 0.001). Gender was significantly associated with overall HCAHPS scores. Male patients were significantly more likely to report higher satisfaction than females (Δβ = 1.4; 95% CI [0.5, 2.31]; *p* < 0.001). Race was significantly associated with overall satisfaction. Black patients also reported higher satisfaction compared to White patients (Δβ = 3.64; 95% CI [2.35, 4.93]; *p* < 0.001). Ethnicity was also a significant predictor, with Hispanic patients on average reporting higher HCAHPS scores (Δβ = 6.17; 95% CI [4.86, 7.48]; *p* < 0.001). Admissions Type was significantly associated with HCAHPS scores. Patients admitted for elective and urgent procedures also reported higher satisfaction than those admitted for emergency procedures (Δβ = 4.38; 95% CI [3.34, 5.42]; *p* < 0.001 and Δβ = 3.10; 95% CI [0.51, 5.69]; *p* = 0.02). Additionally, for each 1-unit increase in CCI, participants had lower satisfaction scores (Δβ = −0.29; 95% CI [−0.4, −0.17]; *p* < 0.001).

The same analysis for the *Communication about Medicines* domain revealed that, on average, patients who watched 50% or more of the educational videos reported higher positive scores compared to those who did not watch the videos [Mean = 65.7 vs 63.6; Δβ = 2.50; 95% CI [0.59, 4.40]; *p* = 0.01]. Additionally, as shown in Table 5, there was no significant difference in the reported scores between those who watched less than 50% and those who did not watch at all [Mean = 66.8 vs. 63.6; Δβ = 3.54; 95% CI [−0.70, 7.79]; *p* = 0.10]. In addition, for each 1-unit increase in age, patients were more likely to report lower satisfaction with communication about medicines, with an estimated decrease of 0.26 (95% CI [−0.33, −0.20]; *p* < 0.001). Gender was also a significant predictor, with Male patients reporting significantly higher satisfaction with medication communication than female patients (Δβ = 3.83; 95% CI [2.02, 5.65]; *p* < 0.001). Race was also a significant predictor, with Black patients reporting higher levels than White patients (Δβ = 8.68; 95% CI [6.05, 11.31]; *p* < 0.001). Ethnicity was also significant. Hispanic patients were significantly more likely to report satisfaction with medication communication (Δβ = 8.20; 95% CI [5.46, 10.94]; *p* < 0.001). Substantially, with every unit increase in Charlson's comorbidity index, participants report lower satisfaction scores (Δβ = −0.27; 95% CI [−0.50, −0.04]; *p* = 0.02).

### 3.3 Readmissions

The readmission analysis comprised 74,052 encounters: 54,477 in which no video was watched, 3131 in which 0–49% of a video was watched, and 16,444 in which 50% or more was watched. No encounter appeared more than once, but a patient admitted on several occasions contributed multiple encounters, and 11,660 of the 74,052 encounters (15.7%) had a medical record number that appeared elsewhere in the dataset. Within individual hospitals, the unadjusted relative reduction in 30-day readmission among encounters in which a video was watched ranged from 19% at the site where the difference was smallest (8.2% versus 10.1%) to 31% at the site where it was largest (7.7% versus 11.2%). Pooled across the system, the unadjusted 30-day readmission rate was 8.2% among encounters in which at least 50% of a video was watched, compared with 11.1% among non-watchers, a relative reduction of approximately 26%.

Among hospital characteristics, the academic medical center, which has the most beds, showed the largest reduction in readmission among encounters in which a video was watched, and the smallest hospital (178 acute care beds) showed the smallest reduction. The hospitals with the largest reductions had the most diverse patient populations and the highest case mix indices.

As shown in Table 6, the findings from the multivariable GEE-GLM logistic regression model showed that patients who watched at least 50% of the videos had 28% lower adjusted odds of 30-day readmission than patients who did not watch the videos (OR = 0.72; 95% CI [0.62–0.84]; *p* < 0.001). Patients with elective admissions had 43% lower adjusted odds of readmission than patients with emergency admissions (OR = 0.57; 95% CI [0.47–0.70]; *p* < 0.001). Additionally, each one-unit increase in the CCI was associated with 11% higher adjusted odds of readmission (OR = 1.11; 95% CI [1.09–1.12]; *p* < 0.001).

As shown in Table 7, the generalized logistic regression for the *Communication about Medicines* domain revealed that patients who watched at least 50% of the videos had 23% lower adjusted odds of readmission than those who did not watch at all (OR = 0.77; 95% CI [0.62, 0.95]; *p* = 0.01). However, the difference between those who watched 0–49% and those who did not watch was not statistically significant. Patients with elective admissions had 41% lower adjusted odds of readmission than those with emergency admissions (OR = 0.59; 95% CI [0.44–0.78]; *p* < 0.001). Race and Ethnicity differences were not significant. Additionally, a higher CCI was associated with higher odds of readmission (OR = 1.11; 95% CI [1.09–1.13]; *p* < 0.001).

### 3.4 Patient comments

Four themes were identified across the free-text comments reviewed. Comments could be assigned to more than one theme, and the number of assignments to each was: pain management and dosing issues, 301; medication understanding, communication and technology quality, 281; discharge planning and medication continuity, 256; and medication errors or concerns, 107. Within the medication understanding, communication and technology quality theme, comments about the video program were largely favorable, with patients writing that they “liked the updates & videos for medications given to patient” and that “the updates were easy to have as reminders and share with family.” Comments in the same theme also identified limits to the program: one patient noted that the videos “were not always about a relevant medication in my particular case,” while adding “otherwise great stuff, please continue the texts,” and another wrote that while they appreciated the videos, they found the medication information in the patient portal “difficult to interpret.” Comments assigned to the medication errors or concerns theme described problems the videos did not resolve, most often around medications at discharge. One patient wrote: “Better communication did not was not sent home with all my prescriptions I need to get well need to speak with someone in reference to why my meds were not provided to take at home very confused need clarification PLEASE HELP” Comments are reproduced as written., or to trim it with ellipses, which would risk softening a complaint. Second, and separately from the manuscript: this patient reported going home without prescriptions and asked for help. Please confirm that comments of this kind were routed through the normal patient-complaint channel at the time, since an editor or reviewer may reasonably ask, and it matters independently of the paper.]

## 4 Discussion

To our knowledge, most research on medication education has focused on the outpatient setting. In the inpatient setting, traditional methods have predominantly involved face-to-face education and written materials. However, these methods of medication education are often expensive, impractical from a staffing perspective, time-consuming, and difficult to scale to large numbers of patients. A digital communication mode may provide a more cost-efficient and scalable alternative. Video messaging services are widely available, and most hospitals have access to such services. Furthermore, videos themselves are often integral to many, if not most, patient education vendor libraries. Indeed, we did not create new content or select new vendors for this study. We were able to connect with two vendors our hospital system already uses for all forms of patient texting and patient education, at no additional cost.

There have been other studies on medication education videos. A recently published systematic review of patient education videos found 12 articles from four countries on medication education interventions that met inclusion criteria.^21^ The systematic review also found that very few studies were well-designed enough to discern the mechanisms underlying behavior change. The findings also suggested that the videos used in previous studies were notably long, ranging from 4 to 60 min, and included up to 112 patients. Moreover, the primary endpoint in those studies of medication education videos focused on increasing patients' understanding and knowledge, as measured by a variety of self-report questionnaires. None of those studies evaluated other endpoints, like patient experience or readmissions. Our study addresses these gaps by examining other endpoints associated with medication videos. It includes a relatively large sample of patients in both the control and intervention cohorts across a large, diverse healthcare system with several hospitals. We found that a digital mechanism for providing patient education is feasible and that watching at least half of the assigned videos was associated with better patient experience, as measured by HCAHPS performance, and lower 30-day readmission rates.

An interesting finding in our study is that hospitals with the highest acuity and most diverse populations arguably performed best in both readmissions and patient experience, whereas less diverse, often more affluent hospitals with higher socioeconomic status patient populations performed modestly. Given that our study is exploratory and not designed to make causal assertions, we cannot draw sound causal inferences or make concrete statements about why these findings occurred. There is some insight, perhaps, in a study by Abed et al.^1^ that identified factors potentially linked to the efficacy of video-assisted patient education: the format in which the information is presented and the complexity of the behavior addressed. They found that patients in audio-visual cohorts demonstrated higher recall. Because we used both written and audiovisual content in our study, and the videos were short and relatively non-complex, we could speculate that they appealed to a wider audience and met the cues and interplay requirements articulated by Abed and colleagues.

Another significant contribution of our study is that prior studies on medication education videos have exclusively focused on patients with specific health conditions, such as rheumatology^10^ or dermatology.^5^ Our intervention was applied to patients in any condition for which the medication was prescribed, thereby increasing the generalizability of this study. Because the videos were sent for any newly administered medication rather than for a nominated condition, the cohort is not restricted by diagnosis. We did not, however, compare the health conditions of video watchers with those of non-watchers, or of patients who were readmitted with those who were not, so we cannot say whether the association we observed is even across conditions or concentrated in some of them. Coding the reason for admission, which we discuss under future directions, would allow that question to be answered.

This study had several noteworthy limitations. The most important limitation was the lack of randomization, which may introduce selection bias. We did not use propensity score matching, weighting or any other quasi-experimental method to address it, relying instead on multivariable adjustment for measured covariates. Adjustment of that kind can only account for the characteristics we observed, so we cannot rule out that the differences we report are partly an artifact of self-selection, and residual confounding cannot be ruled out without randomization. Another limitation was the quality of the EHR documentation, which may have contained data errors. For instance, to record mobile cell phone numbers, patients verbally provide their numbers to registration staff, who then manually enter them into the mobile cell phone field. The data entry could have been incorrect, and videos could have been sent to landlines and entered into the mobile cell field within the EHR. A second limitation is that the vendor limitations constrained us. Specifically, if there was no pre-existing medication video on file in the library, no video was sent to the patient for that specific new medication administration. A third limitation concerns the review and interpretation of free-text patient comments, which were analyzed using a grounded theory approach and are therefore subject to interpretive bias. Although four reviewers coded the comments independently and resolved discrepancies by consensus, some comments could reasonably have been assigned to more than one thematic category. AI-assisted tools were used to support the initial organization of codes, which may have led to preliminary misclassifications, although all final coding decisions and thematic assignments were reviewed and validated by the study team. The themes identified should therefore be read as the predominant patterns among the comments reviewed rather than as an exhaustive account of patient concerns.

A fourth limitation concerns the composition of the comparison group. Because it comprises all survey returns other than those in which a video was watched, it includes encounters in which no video was sent, for example where no video existed in the library for the medication administered, or where no mobile number or consent to text messaging was recorded. Encounters of that kind may differ systematically from encounters in which a video was sent and not opened, and from one another, in ways related to both outcomes. The contrast we report is therefore not a comparison of watchers and non-watchers drawn from a single eligible population, and part of the difference we observe may reflect who was reachable by the program rather than who engaged with it. A fifth limitation is that we could measure readmissions only to facilities within the study health system because our data source was the system's EHR, which captures encounters only at those facilities. A patient readmitted to a hospital outside the system would be recorded as a new admission at that facility and would not appear in our data, so readmission rates in both groups are likely to be underestimated. A sixth limitation is that, as an observational study, we were unable to measure several characteristics that plausibly influence both the decision to watch a video and the outcomes we examined, including patient activation, digital literacy, socioeconomic status, caregiver support, and illness severity beyond what the CCI captures. Residual confounding by these factors cannot be excluded. The higher HCAHPS scores and lower odds of readmission we report should therefore be interpreted as associations rather than effects of the intervention, and prospective or randomized designs will be needed to establish whether the videos themselves change patient experience or readmission rates. The participant flow we are able to report begins at the point of video delivery. The counts of patients screened, started on a new medication, and reachable by text message were not retained in a form that could be reconstructed for this analysis, so the proportion of admitted patients the program reached cannot be established from these data.

One of the most significant limitations of this article, and an area ripe for future research, is the behavioral mechanisms that enhance the efficacy of medication education. It may be that patients who are more active in their healthcare, including watching educational videos, are also more adherent to post-discharge care regimens and therefore less likely to be readmitted. Or patients who are fully engaged and activated in their healthcare are also happier with their hospital experience and therefore report higher HCAHPS scores. Given the exploratory nature of this work, we cannot make definitive claims about which behaviors or attributes influence outcomes. We can only identify associations and note that the literature suggests that activated patients achieve healthier outcomes and report more positive experiences with their healthcare,^7^, ^9^ so there is likely a latent causal attribute that remains undetected.

Another important limitation of the study is its reliance on survey data. We do not have survey weights, and because this is a local hospital survey, the population description and conclusions should not be considered generalizable. Additionally, when studying the local population (not at the national level), adding weights increases variance and does not improve the validity of coefficient estimates.

### 4.1 Future directions

What remains to be seen, and what we suggest as an area for future research, is identifying the behaviors or attributes that manifest among patients who watch videos, and whether and how healthcare systems can promote them. While existing literature shows that patients who are more engaged in general also participate in preventive behaviors, like immunizations and screenings,^8^, ^15^ and are more likely to adhere to treatment,^6^ it is unclear what characteristics predispose someone to watch a video and how that links with their outcomes. One way to examine this is to analyze the patient characteristics and activation levels of those who watch medication education videos versus those who do not. Existing activation scales could be administered to patients, and a taxonomy of characteristics for video-watchers could be developed and applied to higher- and lower-activation participants to determine whether and how activation characteristics and behaviors are associated with video-watching.

Two further questions arise from the data collected here but fell outside the scope of this analysis. First, we did not examine whether videos for particular medication classes or indications were more likely to be watched; describing viewing by drug class or indication would help identify where video education adds most value and where another format may be needed. Second, although admission type was included in our models, we did not code the reason for the index admission. Linking viewing and readmission to the admitting diagnosis or service line would show whether the observed association is concentrated in particular conditions.

Finally, more work is warranted to explore additional safeguards and considerations for privacy and compliance for processes such as pharmacist alert routing, multilingual validation, and HIPAA texting safeguards.

## 5 Conclusion

Our study advances the literature by showing that medication education videos can be scaled to thousands of patients in the inpatient setting within a short time frame and at low cost. In this exploratory, non-randomized analysis, patients who watched at least half of the videos sent to them reported higher HCAHPS scores across multiple domains and had lower 30-day readmission rates than patients who watched none. These are associations; prospective or randomized evaluation is needed before the videos themselves can be said to account for the differences observed.

## CRediT authorship contribution statement

**Courtenay R. Bruce:** Methodology, Investigation, Conceptualization, Writing – original draft. **Ghalib A. Abbasi:** Data curation, Writing – review & editing. **Ashleigh Kamencik-Wright:** Investigation, Writing – review & editing. **Terrell L. Williams:** Investigation, Writing – review & editing. **John Osborn:** Software, Data curation, Writing – review & editing. **Janet Reha:** Data curation, Writing – review & editing. **Claudia Garcia:** Data curation, Writing – review & editing. **April Romero:** Data curation, Writing – review & editing. **Richard Eke:** Data curation, Writing – review & editing. **Tariq Nisar:** Formal analysis, Writing – review & editing. **Farzan Sasangohar:** Supervision, Methodology, Writing – review & editing. **David Putney:** Investigation, Writing – review & editing.

## Declaration of competing interest

The authors do not have any competing interests to report.

## Figures and Tables

**Table:** 

Dimension (Composite)	HCAHPS questions (Abbreviated)
Communication with nurses	- How often did nurses treat you with courtesy and respect? - How often did nurses listen carefully to you? - How often did nurses explain things in a way you could understand?
Communication with doctors	- How often did doctors treat you with courtesy and respect? - How often did doctors listen carefully to you? - How often did doctors explain things in a way you could understand?
Responsiveness of hospital staff	- After pressing the call button, how often did you get help as soon as you wanted? - How often did you get help in getting to the bathroom or in using a bedpan as soon as you wanted?
Communication about medicines	- Before giving any new medicine, how often did staff tell you what it was for? - How often did staff describe possible side effects in a way you could understand?
Discharge information	- Did someone explain what you needed to do during your recovery at home? - Did you get information in writing about what symptoms or health problems to look out for?
Care transition	- Staff took my preferences and those of my family into account when planning care. - I had a good understanding of the things I was responsible for in managing my health. - I clearly understood the purpose for taking each of my medications.
Cleanliness of hospital environment	- How often were your room and bathroom kept clean?
Quietness of hospital environment	- How often was the area around your room quiet at night?
Overall hospital rating	- On a scale from 0 to 10, how would you rate this hospital?
Recommendation of hospital	- Would you recommend this hospital to your friends and family?

Note. Response options for the official HCAHPS items are “Never/Sometimes/Usually/Always” for the frequency items (communication with nurses and doctors, responsiveness, communication about medicines, cleanliness, quietness); “Yes/No” for the discharge information items; “Strongly disagree” to “Strongly agree” for the care transition items; a 0–10 scale for the overall hospital rating; and “Definitely no/Probably no/Probably yes/Definitely yes” for willingness to recommend. The observation-status survey administered at our sites uses the same questions and the same response scales as the official inpatient HCAHPS instrument. Domain scores are top-box percentages: the number of responses in the most positive category divided by the number of responses to that item. Patients who did not answer an item are excluded from its denominator.
